# A systematic review and meta-analysis of the association between fluoride exposure and neurological disorders

**DOI:** 10.1038/s41598-021-99688-w

**Published:** 2021-11-22

**Authors:** Giza Hellen Nonato Miranda, Maria Olímpia Paz Alvarenga, Maria Karolina Martins Ferreira, Bruna Puty, Leonardo Oliveira Bittencourt, Nathalia Carolina Fernandes Fagundes, Juliano Pelim Pessan, Marília Afonso Rabelo Buzalaf, Rafael Rodrigues Lima

**Affiliations:** 1 Laboratory of Functional and Structural Biology, Institute of Biological Sciences, Federal University of Pará (UFPA), Rua Augusto Corrêa nº 1, Belém, PA 66075-110 Brazil; 2 School of Dentistry, Faculty of Medicine and Dentistry, University of Alberta, Edmonton, Canada; 3 Department of Preventive and Restorative Dentistry, School of Dentistry, São Paulo State University (UNESP), Araçatuba, SP Brazil; 4 Department of Biological Sciences, Bauru Dental School, University of São Paulo (USP), Bauru, SP Brazil

**Keywords:** Neurological disorders, Environmental sciences, Neurology

## Abstract

Different studies have suggested that fluoride is related to neurological disorders in children and adolescents, but clinical evidences of which neurological parameters associated to fluoride exposure are, in fact, still controversial. In this way, this systematic review and meta-analysis aimed to show if there is an association between fluoride exposure from different sources, doses and neurological disorders. Terms related to “*Humans*”; “*Central nervous system*”; “*Fluorides*”; and “*Neurologic manifestations*” were searched in a systematic way on *PubMed*, *Scopus*, *Web of Science*, *Lilacs*, *Cochrane* and *Google Scholar.* All studies performed on humans exposed to fluoride were included on the final assessment. A meta-analysis was then performed and the quality level of evidence was performed using the *GRADE* approach. Our search retrieved 4,024 studies, among which 27 fulfilled the eligibility criteria. The main source of fluoride was naturally fluoridated water. Twenty-six studies showed alterations related to Intelligence Quotient (IQ) while only one has evaluated headache, insomnia, lethargy, polydipsia and polyuria. Ten studies were included on the meta-analysis, which showed IQ impairment only for individuals under high fluoride exposure considering the World Health Organization criteria, without evidences of association between low levels and any neurological disorder. However, the high heterogeneity observed compromise the final conclusions obtained by the quantitative analyses regarding such high levels. Furthermore, this association was classified as very low-level evidence. At this time, the current evidence does not allow us to state that fluoride is associated with neurological damage, indicating the need for new epidemiological studies that could provide further evidences regarding this possible association.

## Background

Fluoride (F) has been used as preventive and therapeutic agent in dentistry for over eight decades. It is widely known that its main side-effect (i.e., dental fluorosis) was reported decades prior to the accidental discovery of its caries-preventive effects^1^, further leading to investigations on the mechanisms of action involved, acute and chronic toxicity, as well as its safety and modes of administration. In brief, F can be delivered by community-based strategies (e.g., water, salt and milk fluoridation schemes), as well as by professionally- or self-application methods (e.g., toothpastes, mouthrinses, gels and varnishes), alone or in association^2^, and its use is regarded as safe and cost-effective when administered within the recommended levels^3,4^.

As for community-based methods, water fluoridation is by far the most widely used worldwide, covering ~ 400 million people in 25 countries^5^. It is regarded as a cost-effective method, consisting of the controlled addition of F to the public water supply at concentrations typically ranging from 0.7 to 1.2 mg/L, depending on the mean annual temperature^6^. Water fluoridation was considered as one of the ten greatest public health measures of the twentieth century achievements according to the US Center of Disease Control and Prevention^7^, which is endorsed by several scientific societies, including the World Health Organization (WHO).

Despite the body of evidence attesting the efficacy and safety of water fluoridation, this method has been the subject of heated debate in several parts of the world, questioning legal aspects of the compulsory nature and potential harmful effects. Within this context, a recent systematic review with meta-analysis attempted to demonstrate the relationship between F exposure from the drinking water and intelligence quotient (IQ) impairment, concluding that exposure to water containing high F levels interferes with the child's intelligence development^8^. It is noteworthy, however, that no clear-cut threshold was established to determine which F levels would correspond to each study group, resulting in a wide variability within the control (0.25 to 1.03 mg F/L) and exposed (0.8–11.0 mg F/L) groups, with some overlaps between them. Others reviews were designed to reunite the evidences regarding F developmental neurotoxicity^9,10^ and have highlighted the detrimental effects of high fluoride doses in children exposed by fluoridated water. It is important to highlight that the present study gathered evidences not only from children, but adults exposed to all fluoride sources according to the search strategy. Moreover, we seek to investigate the available evidences about neurological damages in general, not only mnemonic aspects. Also, some of the concentrations included in the control group are not effective for caries control according to the WHO criteria, so that the issue of risks and benefits resulting from exposure to fluoridated water could not be analyzed. Furthermore, the review focused on IQ impairment without considering other neurological disorders that could also potentially be associated with F exposure.

Considering the above, the present systematic review and meta-analysis aimed to investigate the impact of environmental exposure to F from different sources on neurological disorders in humans. For studies that assessed F exposure from water, this review adopted the WHO guidelines to dichotomize between low (0.5 to 1.0 mg F/L) and high (above 2 mg F/L) exposure, allowing the discussion of doses safety of water fluoridation.

## Methods

### Protocol and registration

This systematic review was registered in PROSPERO database, under CRD number 42017067234. A review was performed according to Moher, Liberati^11^, followed as recommendations by the Preferred Reporting Items for Systematic Reviews and Meta-analyses (PRISMA) statement.

### Eligibility criteria and search strategy

This review was designed using the PECO strategy and based on it, observational studies in humans (P) exposed to high concentrations of F (E) and low concentrations (C) in which the associations between F and neurological damage (O) were investigated. Case reports, descriptive studies, review articles, opinion articles, technical articles, guidelines, as well as animal and in vitro studies were disregarded.

The study was based on the question: "Can chronic F exposure be associated with neurological damage?" The searches were performed in January 2021, with no restrictions on the date of publication and the language of the studies. The electronic databases used were: *PubMed*, *Scopus*, *Web of Science*, *Lilacs*, *Cochrane* and *Google Schoolar.* The MeSH terms used were: “*Humans*”; “*Central nervous system*”; “*Nervous system*”; “*Fluorine*”; “*Fluorides*”; “*Fluorine Compounds*”; “*Fluoride Poisoning*”; “*Neurobehavioral manifestations*”; “*Nervous System Disease*”; “*Neurologic manifestations*”; “*Intelligence*”*.* All MeSH keywords and search strategy were adapted according to the specifics of each database, as represented in Table A.1.

After the search stage, an alert was registered in each database for weekly notification of new studies that fit the vested strategy. All citations were entered into a bibliographic reference manager and duplicate studies were excluded, either automatically or manually (EndNote®, v. X7, Thomson Reuters, Philadelphia, USA). The search, study selection, risk of bias and data extraction stages were performed independently by two evaluators (G.H.N.M; M.O.P.A.) and checked by a third evaluator in case of disagreement (R.R.L).

Then, the study selection was made based on the title and abstract of articles and then by full-text analysis according to the recommended eligibility requirements. Reference lists of included studies were also evaluated for study selection.

### Data extraction and assessment of methodological quality and risk of bias

From the included articles, data regarding the year of publication, study design, participant characteristics (origin and sample size), mean age, F concentration measurement parameters, diagnostic criteria for assessment of cognitive performance, results and statistical analysis were extracted and tabulated. In case of doubts about the methodology, lack of data in the studies and inability to find full articles, the authors were contacted via email with a weekly message for three consecutive weeks.

To assess the methodological quality and risk of bias, the checklist of Fowkes and Fulton^12^ was applied. This checklist has domains that relate to study and sample design; control group characteristics; quality of measures and results; and distorted integrity and influences.

After evaluating each criterion, a (++) sign was assigned for major study problems or (+) for minor problems to assess whether the methods are adequate to produce consistent and valid information, as well as whether the results offered the expected effects. In items where the question was not applicable to the type of study, it was assigned the acronym NA (not applicable). "No problem" has been assigned the sign (0). The evaluation for each domain was standardized by the examiners and is described in Table A.2.

After detailed evaluation of the methods and results, the studies were analyzed to verify the possibility of "skewed results", "confusions" and "random occurrence". To determine the value of the study, three summary questions were answered: "Were the results biased?"; "Are factors of confusion or distortion present?" and "Is there a possibility that the results came about by chance?" "YES" and "NO" answers were given. If the answer is NO in the three questions, the article is considered reliable, with low risk of bias.

### Quantitative synthesis (meta-analysis)

The studies data were analyzed using Review Manager software (Review Manager v. 5.3, The Cochrane Collaboration; Copenhagen, Denmark) to evaluate if Chronic exposure to F is associated with neurological deficit. In all analyses, only studies with low risk of bias were included.

A meta-analysis was performed to compare the percentage of low IQ with high and low chronic exposure to F. Previously, each study classified the F levels as low or high with heterogeneous concentrations. Then, for the meta-analysis we decided to classify the studies according to the WHO guidelines that consider optimal levels between 0.5–1.0 mg/L (low levels) and > 2 mg/L, as higher levels for water fluoridation^13,14^. The number of people with low IQ and the total number of participants in each case group (high fluoride) and control group (low fluoride) were included to calculate the odds ratio with a 95% confidence interval (CI).

The heterogeneity among studies was tested using I^2^ index (p-value < 0.05 was considered statistically significant). A fixed and random effects models were used in the analyses of the studies. The final choice regarding effects model was performed based on I^2^ index^16^. The forest plots were generated for each analysis and an alpha of 0.05 was adopted as the cut-off point for significance.

The publication bias was assessed through a comprehensive analysis of Egger’s test, and Funnel Plot Visual interpretation^17^. A p-value < 0.05 indicated a likely publication bias across the studies. The Jamovi statistical software (version 1.6, Sydney, Australia) was used to generate figures and to run the test.

A sensitivity analyses was used to explore the influence of each study in the pooled meta-analysis or publication bias results. This analysis was adopted in case of substantial or considerable (50 to 100%) heterogeneity, or significant publication bias (p < 0.05). This evaluation was performed by manually omitting one study at time, one by one, and verifying its impact in the final results^15^.

### Level of evidence assessment–GRADE

The level of evidence was determined using the Grading of Recommendations Assessment, Development and Evaluation (*GRADE*) approach. This tool provides a structured process for developing and presenting evidence summaries that measure the quality of evidence to confirm or reject hypotheses in systematic reviews^18^.

GRADE has four *levels* of *evidence* –decreasing from low to very low, moderate, and high; depending on whether issues such as risk of bias, inconsistency, inaccuracy and publication bias are severe, very serious or not serious. Although, observational studies begin as poor-quality evidence, the level can increase from low to high if the magnitude of the effect is large or very large^19^.

### Consent for publication

All the authors are in accordance with the publication.

## Results

### Search results

**Figure 1:**
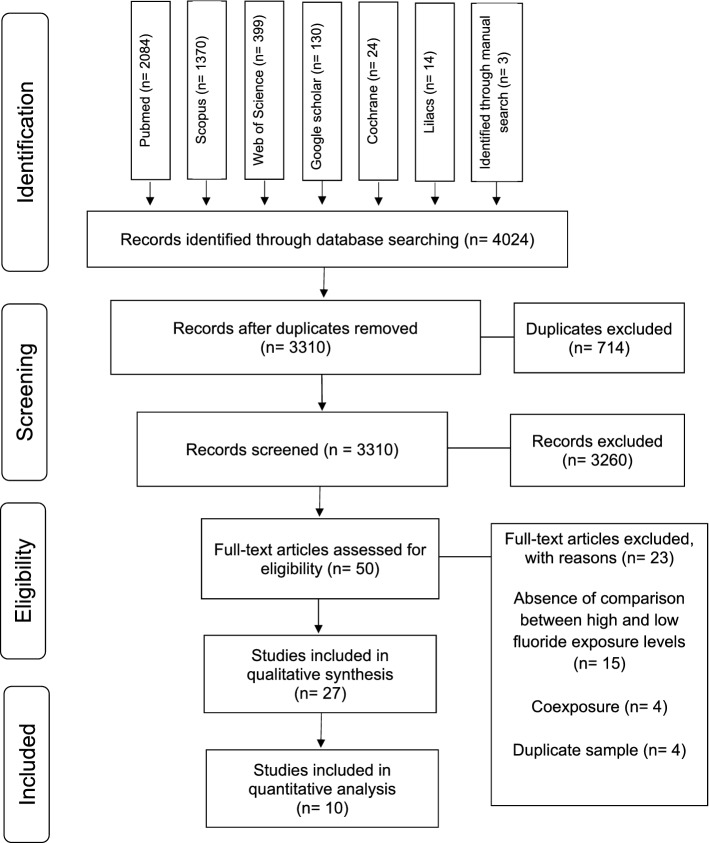
Flow diagram of databases searched according to PRISMA guidelines. PRISMA, Preferred Reporting Items for Systematic Review and Meta-Analysis.

Based on the database searches, 4,024 studies were found. Three studies were included after manually searching in the reference lists^20–22^. After the removal of duplicate studies (714), 3,310 articles remained and were analyzed by title and abstract according to the eligibility criteria. A total of 3,260 were excluded, and 50 studies remained for full text reading. Fifteen studies were excluded because, when assessing IQ, they did not compare between high and low F concentrations, four contained co-exposure of F and other concomitant elements, and four used the same sample from studies included in this systematic review (Table A.3). Thus, 27 studies were elected, which underwent quality assessment of the risk of bias. The summary of the selection process is shown in Fig. 1.

### Characteristics of the studies

The 27 included studies were characterized as observational, cross-sectional type, among which 26 were analytical studies, and one was descriptive^23^. The age group investigated included individuals from 6 to 18 years of age. Most of the articles evaluated F exposure due to ingestion of naturally fluoridated water. Only one study analyzed populations exposed to F by burning coal^24^.

The F concentrations in drinking water categorized as low exposure in the selected studies ranged from 0.19 ppm^25^ to 2.01 ppm^26^, while high doses ranged from 1.5 ppm^23,27^ to 8.3 ppm^28^. Some studies considered a third intermediate category^23,29–33^, which ranged from 0.5 ppm^30^ to 3.1 ppm^33^. One study classified exposed groups according to four concentration levels, ranging from < 0.7 ppm to > 4.0 ppm^21^. One study did not provide high and low dose reference concentrations^20^ and the study developed with F exposure from coal burning^24^ reported only the content of F related to high exposure (0.0298 mg/m^3^).

Regarding the source of sample used for the estimation of F exposure, most of the studies evaluated the drinking water alone^20,21,24,25,29–31,34–36^, followed by measurement in both drinking water and urine of participants^20,22,26–28,37–41^, and in the air^24^. Some studies did not quantify the F levels, however determined the concentration from data available from national databases or electronic addresses^32,42,43^. Three studies did not report the process used, nor the source consulted to establish F exposure^23,44,45^, and mentioned the use of conventional chemical tests only without specifying the method for F^46^.

In relation to the parameters of cognitive assessment, in 26 studies the IQ was used to estimate a comparative intellectual and stabilizing capacity between the high and low groups, whereas one study^23^ evaluated neurological manifestations such as headache, insomnia, lethargy, polydipsia and polyuria. The tests applied for IQ evaluation varied among the studies, being the "Raven's Standard Progressive Matrices test"^20,21,27,29–31,34,38^ and the "Standardized Chinese Test"^22,28,37,40,41,44,46^ the most used, followed by "Raven's Color Progressive Matrices"^25,32,33,43^, "Stanford-Binet Intelligence Scale"^26,39^, "Chinese Binet IQ Test"^24^, "Prueba Raymond B Cattell"^35^, "Wechsler Preschool Guidelines and Primary Intelligence Scale (WPPSI)"^36^, "Rui Wen Prueba Handbook"^45^ and "Form Board Test"^42^. The descriptive study^23^ used as a tool for data collection, interviews with questionnaires prepared by qualified professionals.

In the analysis of results, 23 studies showed a statistical difference between exposure to high and low doses of F. In three studies a comparison of intellectual skill among the groups exposed to high and low F concentrations was not statistically significant^30,34,46^. The descriptive study^23^ reported the presence of alterations related to neurological manifestations in some group in high dose exposure (1.5–6.4 ppm). Table 1 shows details of all the characteristics of the included studies.

**Table 1:** Data extraction from included studies.

Author, (year)	Study design	Participants			Case evaluation		Statistical analysis	Results	Risk of bias
		Source of sample	Sample size and levels of fluoride exposure	Age (years)	Neurological assessment	Fluoride levels measurement			
Aravind et al., (2016)	Cross-sectional	Mastihalli, Banavara and Virajpet, Hassan, India	(n = 180) 60: High (> 3 ppm) 60: Medium (1.2–2 ppm) 60: Low (< 1.2 ppm)	10–12	Raven's Standard Progressive Matrices test	Evaluation by ion selective electrode method in water samples	Analysis of variance (ANOVA), Student's t-test, Kruskal–Wallis ANOVA and Spearman's rank correlation coefficient	The mean IQ level was more in the region with medium fluoride concentration in drinking water (56.68 ± 14.51) compared to areas with low fluoride concentration (41.03 ± 16.36) and high fluoride concentration (31.59 ± 16.81); p < 0.0001	Low
Chen et al., (1991)	Cross-sectional	Biji village and Jiaobei village, Linyi County, Shanxi Province, China	(n = 640) 320: High (4.55 ppm) 320: Low (0.89 ppm)	7–14	Chinese Standardized Raven Test	N/I	t‑test	The average IQ of children in lower fluoride área (104.03 ± 14.96) was significantly higher than that of in the higher fluoride (100.24 ± 14.52); p < 0.01	Low
Eswar et al., (2011)	Cross-sectional	Davangere district, Karnataka, India	(n = 133) 68: High (2.45 ppm) 65: Low (0.29 ppm)	12–14	Raven's Standard Progressive Matrices test	Evaluation by ion selective electrode method in water samples	Chi-square and Z tests	There were no significant differences in IQ score of children living in high drinking water fluoride region (86.3 ± 12.8) and children living in low drinking water fluoride region (88.8 ± 15.3); p = 0.30	High
Guo et al. (1991)	Cross-sectional	Xinshao County, Hunan Province, China	(n = 121) 60: High (0.0298 mg/m^3^) 61: Low (N/I)	7–13	Chinese Binet IQ Test	N/I	Correlation analysis	In the high fluoride area, the correlation co-efficient r = –0.25 (p<0.05), and for the control area r = –0.07 (p>0.05), for the two combined r = –0.205 (p<0.05). These results indicate that there is a negative correlation between serum fluoride and IQ, and that the correlation is greater within the high fluoride group. The average IQ of the endemic area children was 76.7, and the control group children had average IQs of 81.4; when compared, the difference is statistically significant; p < 0.05	Low
Hong et al. (2001)	Cross-sectional	Wukang, Boxing, Zouping, Shangdong Province, China	(n = 117) 85: High (2.90 ppm) 32: Low (0.75 ppm)	8–14	Chinese Standardized Raven Test	Evaluation by conventional chemical assay methods	t-test and Chi-squared	There is no significant difference between the high fluoride (80.58 ± 2.28) and control areas (82.79 ± 8.98); p > 0.05	Low
Karimzade et al., (2014)	Cross-sectional	West Azerbaijan, Iran	(n = 39) 19: High (3.94 ppm) 20: Low (0.25 ppm)	9–12	Raymond B Cattell test	Evaluation by SPADNS colorimetric method in water samples	Unpaired t test and chi-squared testing	The mean IQ of children living in high drinking water fluoride region (81.21 ± 16.17) was lower than that of children living in low drinking water fluoride region (104.25 ± 20.73); p=0.0004	Low
Khan et al., (2015)	Cross-sectional	Asoha block in district Unnao and Tiwariganj block in district Lucknow of Uttar Pradesh, India	(n = 429) 214: High (2.41 ppm) 215: Low (0.19 ppm)	6–12	Raven’s Coloured Progressive Matrices (RCPM)	Evaluation by ion selective electrode method in water samples	Chi-squared test, ANOVA, Post-Hoc and Spearman’s rank correlation	Difference in IQ grade of children from different locations was found to be statistically significant (p < 0.001).	Low
Kundu et al., (2015)	Cross-sectional	Najafgarh and Defence Colony, Delhi, India	(n = 200) 100: High (N/I) 100: Low (N/I)	8–12	Ravens Standardized Progressive Matrices Test	Evaluation by ion selective electrode method in water samples	Independent *t*‑test	Comparison of mean IQ of children in high (76.20 ± 19.10) and low F (85.80 ± 18.85) areas showed a significant difference; p = 0.013	High
Lu et al., (2000)	Cross-sectional	Tianjin Xiqing District, China	(n = 118) 60: High (3.15 ± 0.61 ppm) 58: Low (0.37 ± 0.04 ppm)	10–12	Chinese Combined Raven’s Test, Copyright 2 (CTR-C2)	Evaluation by ion selective electrode method in water and urine samples	Fisher’s exact test, Welch’s alternate t-test, the rank sum test, and multiple regression analysis	The IQ of high fluoride area was significantly lower (92.27 ± 20.45) than that of the children in the low fluoride area (103.05 ± 13.86); p < 0.005	Low
Nagarajappa et al., (2013)	Cross-sectional	Mundra and Bhuj, Kutch District, Gujarat, India	(n = 100) 50: High (2.4–3.5 ppm) 50: Low (0.5 ppm)	8–10	Seguin Form Board Test	Based on Water and Sanitation Management Organization, Gujarat	Independent student *t*-test	Mean IQ scores were found to be significantly higher among children living in low fluoride region (30.45 ± 4.97) than those living in high fluoride region (23.20 ± 6.21); p < 0.05	Low
Poureslami et al., (2011)	Cross-sectional	Koohbanan and Baft, Kerman Province, Iran	(n = 120) 60: High (2.38 ppm) 60: Low (0.41 ppm)	7–9	Raven's Progressive Matrices Intelligence Test	Evaluation by ion selective electrode method in water and urine samples	t test and Mann–Whitney test	The mean IQ of children living in high fluoride region (91.37 ± 16,63) was significantly lower than the average IQ of children living in low fluoride region (97.80 ± 15.95); p = 0.028	Low
Qin et al., (2008)	Cross-sectional	Jing County, Hubei Province, China	(n = 447) 141: High (2.1–4.0 ppm) 159: Medium (0.5–1.0 ppm) 147: Low (0.1–0.2 ppm)	9–10	Raven's Standard Progressive Matrices test	Evaluation by ion selective electrode method in water samples	N/I	The difference between the high and low groups exposed was not statistically significant; p > 0.05	High
Razdan et al., (2017)	Cross-sectional	Raya, Farah and Charora; Mathura district, Uttar Pradesh, India	(n = 219) 69: High (2.99 ppm) 75: Medium (1.70 ppm) 75: Low (0.60 ppm)	12–14	Raven's Progressive Matrices Test	Evaluation by ion selective electrode method in water samples	Independent t test, One way analysis of variance, and post hoc analysis and Chi-square test	Comparison between all the groups showed the mean IQ scores in low (38.60 ± 6.33), medium (18.94 ± 4.38), and high (13.94 ± 5.13) fluoride regions a statistically significant difference; p < 0.001	Low
Saxena et al., (2012)	Cross-sectional	Karera Block, Shivpuri district and Parwaliya village, Bhopal district, Madhya Pradesh state, India	(n = 170) 120: High (≥ 1.5 ppm) 50: Low (< 1.5 ppm)	12	Raven's Standard Progressive Matrices	Evaluation by ion selective electrode method in water and urine samples	ANOVA One Way	Comparison of mean IQ of children in high (4.17) and low (3.16) fluoride area showed a significant difference; p = 0.000	Low
Sebastian et al., (2015)	Cross-sectional	Nerale, Belavadi, Naganahall, Mysore district; Carnataca, India	(n = 405) 135: High (2.20 ppm) 135: Medium (1.20 ppm) 135: Low (0.40 ppm)	10–12	Raven’s Coloured Progressive Matrices (RCPM)	Based on Rajiv Gandhi National Rural Drinking Water Program (RGNRDWP)	Analysis of variance (ANOVA), post-hoc test and binary logistic regression	The mean IQ scores for children with normal (88.6 ± 14.01) and low (86.37 ± 13.58) fluoride content were significantly higher than high fluoride level (80.49 ± 12.67); p < 0.01	Low
Seraj et al., (2012)	Cross-sectional	Babur, Panjarlu, Dizaj, Small Donalau and Large Donalau; Makoo, Iran	(n = 293) 91:High (5.2 ± 1.1 ppm) 106: Medium (3.1 ± 0.9 ppm) 96:Low (0.8 ± 0.3 ppm)	6–11	Raven’s Color Progressive Matrices (RCPM)	Evaluation by SPADNS colorimetric method in water samples	ANOVA, Post Hoc test and Kruscal-Wallis	IQ scores for children with low fluoride (97.77 ± 18.91) were significantly higher than the medium (89.03 ± 12.99) and high (88.58 ± 16.01) fluoride level; p = 0.001	Low
Sharma et al., (2009)	Cross-sectional	Sanganer Tehsil, India	(n = 1145) 418: High (1.5–6.4 ppm) 355: Medium (1.0–1.5 ppm) 372: Low (< 1.0 ppm)	12–18	Interviewed (questionnaire) for neurological manifestations (Headache Insomnia Lethargy Polyuria Polydipsia)	N/I	Descriptive analysis	There were no neurological manifestations in children in the low and medium F villages, whereas, in the high F villages, 9.48% of the children had headache, 1.21% had insomnia, and 3.23% exhibited lethargy. There were no cases of polyuria or polydipsia among the children in any of the villages	High
Shivaprakash et al., (2011)	Cross-sectional	Bagalkot taluk and Hungund taluk, India	(n = 160) 80: High (2.5–3.5 ppm) 80: Low (< 0.5 ppm)	7–11	Raven’s Coloured Progressive Matrices	Based on indiawaterportal.org	*t*‑test	The average IQ of children in lower fluoride area (76.3625 ± 20.8431) was significantly higher than that of in the higher fluoride (66.6250 ± 18.0908); p = 0.0019	Low
Sudhir et al. (2009)	Cross-sectional	Nalgonda District, Andhra Pradesh, India	(n = 1000) 247: Level 1 (< 0.7 ppm) 243: Level 2 (0.7–1.2 ppm) 267: Level 3 (1.3–4.0 ppm) 243: Level 4 (> 4.0 ppm)	13–15	Raven's standard progressive matrices	Evaluation by ion selective electrode method in water samples	Chi-square test and Spearmen's rank correlation	Chi-aquare test was used to test the association among the different fluoride levels with IQ scores, and the Spearman's rank correlation was used to measure the relationship between the two variables. The results showed a statistically significant inverse association between both variables (p < 0.001).	Low
Trivedi et al., (2007)	Cross-sectional	Chandlodia, Ahmedabad and Sachana, Sanand district of Gujarat, India	(n = 190) 89:High (5.55 ± 0.41 ppm) 101:Low(2.01 ± 0.09 ppm)	12–13	Questionnaire standardized with 97% reliability rate in relation to the Stanford-Binet Intelligence Scale	Evaluation by ion selective electrode method in water and urine samples	Student’s t test	The mean IQ score of the high F area was significantly lower (91.72 ± 1.13) than that of the lower F area (104.44 ± 1.23). p < 0.001	Low
Trivedi et al., (2012)	Cross-sectional	Baroi, Chhasara, Gundala, Mundra, Pragpar, and Zarpara; Kachchh, Gujarat, India	(n = 84) 34:High (2.3 ± 0.87 ppm) 50:Low (0.84 ± 0.38 ppm)	11–13	Questionnaire standardized with 97% reliability rate in relation to the Stanford-Binet Intelligence Scale	Evaluation by ion selective electrode method in water and urine samples	Paired sample T test	The average IQ level of schoolchildren from the low F villages was (97.17 ± 2.54), which is significantly higher (p ≤ 0.001) than (92.53 ± 3.13) of schoolchildren from the high F villages; p ≤ 0.001	High
Wang et al. (2007)	Cross-sectional	Shanxi Province, China	(n = 449) 253: High (8.3 ± 1.9 ppm) 196: Low (0.5 ± 0.2 ppm)	8–12	Combined Raven's Test The Rural in China (CRT-RC)	Evaluation by ion selective electrode method in water and urine samples	*t*‑test	Comparison of mean IQ of children in high (100.5 ± 15.8) and low F (104.8 ± 14.7) areas showed a significant difference; p < 0.05	Low
Wang et al., (2008)	Cross-sectional	Shehezi, Xinjiang Province, China	(n = 230) 147: High (> 1.0 ppm) 83: Low (≤ 1.0 ppm)	4–7	Wechsler Preschool and Primary Scale of Intelligence (WPPSI) guidelines	Evaluation by ion selective electrode method in water samples	*t*‑test	There was a significant difference in IQ in the endemic area of fluoride concentration (95.64 ± 14.34) compared to the control area (101.22 ± 15.84); p < 0.05	High
Wang et al., (2006)	Cross-sectional	Yuncheng City, Shanxi, China	(n = 368) 202: High (5.54 ± 3.88 ppm) 166: Low (0.73 ± 0.28 ppm)	8–12	Combined Raven’s Test for Rural China (CRT-RC)	Evaluation by ion selective electrode method in water and urine samples	*t*‑test	The IQ in the control group (111.55 ± 15.19) were higher than those of the high fluoride area (107.46 ± 15.38), and the difference was statistically significant, p < 0.01	High
Xiang et al., (2003)	Cross-sectional	Wamiao, Xinhuai, Jiangsu Province, China	(n = 512) 222: High (2.47 ± 0.79 ppm) 290: Low (0.36 ± 0.15 ppm)	8–13	Combined Raven’s Test for Rural China (CRT-RC)	Evaluation by ion selective electrode method in water and urine samples	*t*‑test	The mean QI score of high F village (92.02 ± 13.00) was found to be lower than the mean QI score of low F village (100.41 ± 13.21); p < 0.01	Low
Yu et al., (2018)	Cross-sectional	Tianjin, China	(n = 2886) 1250: High (2.00 ± 0.75 ppm) 1636: Low (0.50 ± 0.27 ppm)	7–13	Combined Raven's Test–The Rural in China (CRT-RC2)	Evaluation by ion selective electrode method in water and urine samples	Student's t-test or Wilcoxon test was used to compare the difference of continuous variables, and Chi-square test was applied to compare the difference of categorical variables	The average IQ score was 107.4 ± 13.0 in the normal-fluoride exposure group, which was statistically higher than the mean level of 106.4 ± 12.3 in the high fluoride exposure group; p = 0.036	Low
Zhao et al., (1996)	Cross-sectional	Sima village, Shanxi and Xinghua village, China	(n = 320) 160: High (4.12 ppm) 160: Low (0.91 ppm)	7–14	Rui Wen Test Manual	N/I	N/I	There was a significant difference in IQ in the endemic area of fluoride concentration (97.32 ± 13.00) compared to the control area (105.21 ± 14.99); p < 0.02	High

IQ, Intelligence Quotient; F, fluoride; N/I, no information; SPADNS (sulfo phenylazo dihydroxy naphthalene disulfonic acid).

### Risk of bias analysis

The quality of the studies was assessed based on risk of bias, confounding factors, and random occurrence. Eight studies were considered of low methodological quality and were classified as high risk of bias^20,22,23,30,34,36,39,45^. The other 19 articles were classified as low risk of bias and, despite having some problems, they were not serious enough to be classified as high risk of bias. In the "Study sample representative" domain, the problem items were the "Sampling method", "Sample size" and "Entry criteria/exclusion". In the "Sampling method", nine studies presented major problems (++) mainly related to the convenience sample. In the item "Sample size", two articles presented major problems, because they did not make a sample calculation and the sample was smaller than 50 participants. In the entry criteria/exclusion section, only two studies presented a minor problem due to co-exposure to arsenic and iodine.

For "Control group acceptable", the item "Definition of controls" presented two articles with minor problems (+) because they did not report the F concentration of the control group. Regarding "Matching/Randomization", nine studies did not mention randomization, but did the matching, being considered as a minor problem (+). However, two articles did not mention randomization or pairing, being considered as a major problem (++).

The domain "Quality of measurements and outcomes", the item with the most serious issues was the "Blindness", as 18 studies did not adopt any kind of blinding, followed by "Quality control", with eight studies that did not describe the measurement method. Table 2 presents the risk assessment of bias of the 27 eligible articles.

**Table 2:** Quality assessment of the studies included in the review.

Guideline	Checklist	Aravind et al., 2016	Chen et al., 1991	Eswar et al., 2011	Guo et al., 1991	Hong et al., 2001	Karimzade et al., 2014	Khan et al., 2015	Kundu et al., 2015	Lu et al., 2000	Nagarajappa et al., 2013	Poureslami et al., 2011	Qin et al., 2008	Razdan et al., 2017	Saxena et al., 2012	Sebastian and Sunitha, 2015	Seraj et al., 2012	Sharma et al., 2009	Shivaprakash et al., 2011	Sudhir et al., 2009	Trivedi et al., 2007	Trivedi et al., 2012	Wang et al., 2007	Wang et al., 2008	Wang et al., 2006	Xiang et al., 2003	Yu et al., 2018	Zhao et al., 1996
Study design appropriate to objectives?	Objective common design																											
	Prevalence Cross-sectional																											
	Prognosis Cohort																											
	Treatment Controled trial																											
	Cause Cohort, case-control, cross-sectional	0	0	0	0	0	0	0	0	0	0	0	0	0	0	0	0	0	0	0	0	0	0	0	0	0	0	0
Study sample representative?	Source of sample	0	0	0	0	0	0	0	0	0	0	0	0	0	0	0	0	0	0	0	0	0	0	0	+	0	0	0
	Sampling method	0	0	++	0	0	0	++	++	0	0	0	0	0	0	++	++	++	0	0	++	++	0	++	0	0	0	0
	Sample size	0	+	+	0	+	++	+	+	+	+	0	0	0	+	+	+	+	+	0	+	++	0	+	+	0	0	+
	Entry criteria/exclusion	0	0	0	0	+	0	0	0	0	0	0	0	0	0	0	0	0	0	0	0	0	+	0	0	0	0	0
	Non-respondents	0	0	0	0	0	0	0	0	0	0	0	0	0	0	0	0	0	0	0	0	0	0	0	0	0	0	0
Control group acceptable?	Definition of controls	0	0	0	+	0	0	0	0	0	0	0	0	0	0	0	0	0	0	0	0	0	0	0	+	0	0	0
	Source of controls	0	0	0	0	0	0	0	0	0	0	0	0	0	0	0	0	0	0	0	0	0	0	0	0	0	0	0
	Matching/randomization	+	0	+	0	+	0	0	+	0	0	0	+	0	0	++	+	+	0	0	+	+	0	++	0	0	0	0
	Comparable characteristics	0	0	0	0	0	0	0	0	0	0	0	0	0	0	0	0	0	0	0	0	0	0	0	0	0	0	0
Quality of measurements and outcomes?	Validity	0		0	0	0	0	0	0	0	0	0	0	0	0	0	0	++	0	0	0	0	0	0	0	0	0	0
	Reproducibility	0	0	0	0	0	0	0	0	0	0	0	0	0	0	0	0	+	0	0	0	0	0	0	0	0	0	0
	Blindness	++	++	++	0	++	++	++	++	0	++	0	0	0	++	0	0	++	++	++	0	++	++	++	++	0	++	++
	Quality control	0	+	0	+	0	0	0	+	0	+	+	++	0	0	0	0	+	+	0	0	0	0	0	0	0	0	++
Completeness	Compliance	0	0	0	0	0	0	0	0	0	0	0	0	0	0	0	0	0	0	0	0	0	0	0	0	0	0	0
	Drop outs	0	0	0	0	0	0	0	0	0	0	0	0	0	0	0	0	0	0	0	0	0	0	0	0	0	0	0
	Deaths	NA	NA	NA	NA	NA	NA	NA	NA	NA	NA	NA	NA	NA	NA	NA	NA	NA	NA	NA	NA	NA	NA	NA	NA	NA	NA	NA
	Missing data	0	0	0	0	0	0	0	0	0	0	0	0	0	0	0	0	0	0	0	0	0	0	0	+	0	0	0
Distorting influences?	Extraneous treatments	0	0	0	0	0	0	0	0	0	0	0	0	0	0	0	0	0	0	0	0	0	0	0	0	0	0	0
	Contamination	NA	NA	NA	NA	NA	NA	NA	NA	NA	NA	NA	NA	NA	NA	NA	NA	NA	NA	NA	NA	NA	NA	NA	NA	NA	NA	NA
	Changes over time	0	0	0	0	0	0	0	0	0	0	0	0	0	0	0	0	0	0	0	0	0	0	0	0	0	0	0
	Confounding factors	0	0	0	0	0	0	0	0	0	0	0	0	0	0	0	0	0	0	0	0	0	0	0	0	0	0	0
	Distortion reduced by analysis	0	0	0	0	0	0	0	0	0	0	0	0	0	0	0	0	0	0	0	0	0	0	0	0	0	0	0
Summary questions	Are the results erroneously biased in certain direction?	No	No	Yes	No	No	No	No	Yes	No	No	No	Yes	No	No	No	No	Yes	No	No	No	Yes	No	Yes	Yes	No	No	Yes
	Confounding:																											
	Are there any serious confusing or other distorting influences?	No	No	Yes	No	No	No	No	Yes	No	No	No	Yes	No	No	No	No	Yes	No	No	No	Yes	No	Yes	Yes	No	No	Yes
	Chance:																											
	Is it likely that the results occurred by chance?	No	No	Yes	No	No	No	No	Yes	No	No	No	Yes	No	No	No	No	Yes	No	No	No	Yes	No	Yes	Yes	No	No	Yes

### Level of evidence

The assessment of the level of certainty of the evidence was conducted through a narrative synthesis following the GRADE parameters for systematic reviews. The level of evidence of the studies was very low, both for studies evaluating IQ impairment and for the only study assessing other neurological manifestations, due to observational nature of the study protocol, as well as due to methodological inaccuracy. For the studies that evaluated IQ impairment, a serious risk of bias was observed. Regarding the study evaluating neurological manifestations other than IQ impairment, it also presented a highly suspicious publication bias, given that the measurement of these manifestations was done by the application of a questionnaire with unknown information about validation and without precise details for their reproduction.

Although, a narrative synthesis does not provide precise estimates, nor measure of effects, it was concluded that the level of evidence of the studies taken together is not strong enough to affirm that the high F exposure may produce a neurological damage in children. Results are represented in Table 3.

**Table 3:** GRADE evidence profile table.

Certainty assessment							Impact	Certainty	Importance
No. of studies	Study design	Risk of bias	Inconsistency	Indirectness	Imprecision	Other considerations			
**Alterations on the Intelligence Quotient (assessed with: Different validated tests to measure IQ)**									
26	Cross-sectional	Not serious	Not serious	Not serious	Serious^a^	None	The IQ was assessed in 9930 patients. Three studies did not present significant differences between the group exposed to high fluoride and the control group; 24 studies showed significant changes for the IQ score (Lower IQ scores for High Fluoride Exposures—1.5 to 8.3 ppm)	⨁◯◯◯ VERY LOW	CRITICAL
**Neurological Manifestations (assessed with: Questionnaire for neurological manifestations (Headache, Insomnia, Lethargy, Polyuria, Polydipsia))**									
1	Cross-sectional	Not serious	Not serious	Not serious	Serious^a^	Publication bias strongly suspected^b^	The neurological manifestation was assessment in 1145 patients. There were no neurological manifestations in children living in villages with low fluoride exposure; in villages with high exposure, 9.48% of the children had headache, 1.21% insomnia and 3.23% lethargy	⨁◯◯◯ VERY LOW	CRITICAL

^a^Narrative synthesis was conducted, non-precise estimates and effects not estimated. Eswar et al., 2011; Kundu et al. 2015; Qin et al., 2008; Sharma et al., 2009; Trivedi et al., 2012; Wang et al., 2008; Wang et al., 2006 and Zhao et al., 1996; ^b^ Non-validated questionnaire, and has no specificity.

### Quantitative analysis

**Figure 2:**
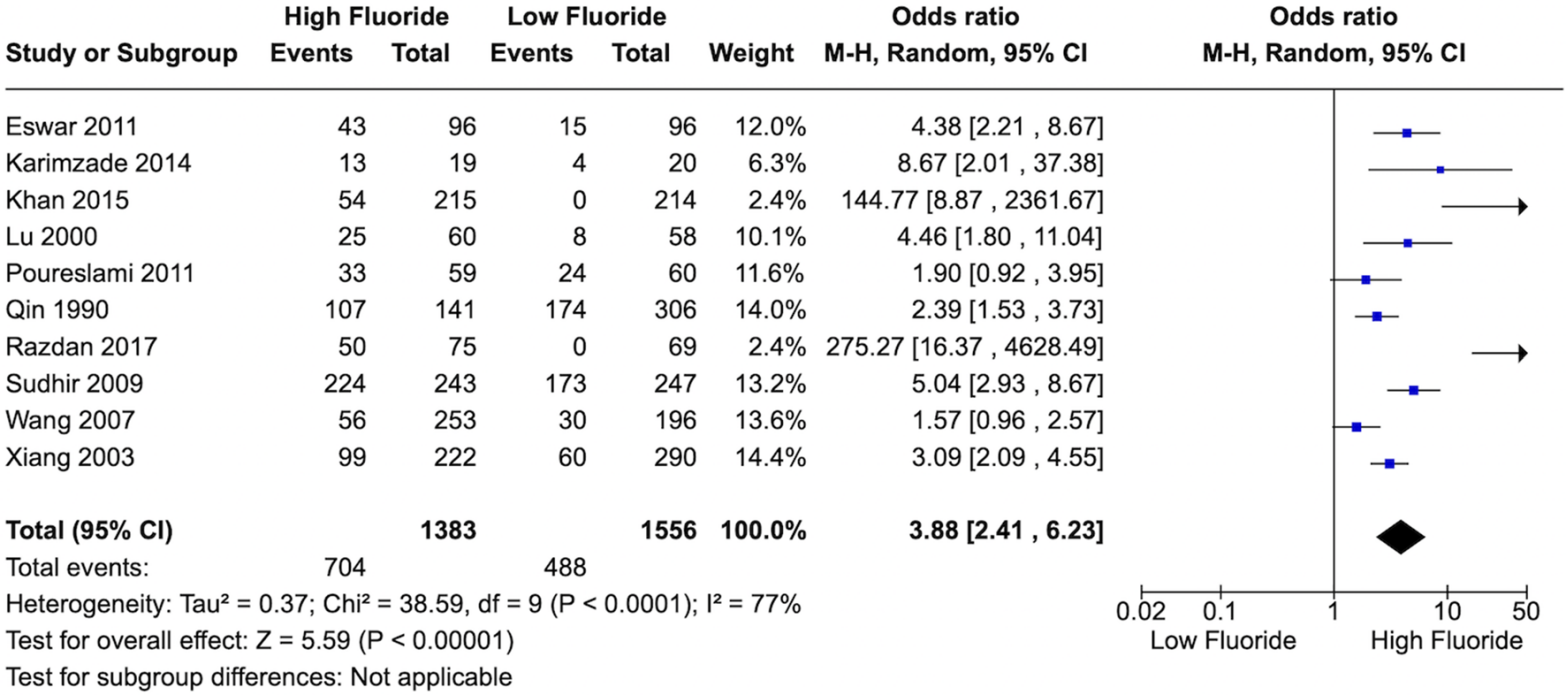
Forest plot of meta-analysis for ten studies (I^2^ = 77%). The association between chronic exposure to fluoride and cognitive deficit. CI, confidence interval; M-H, Mantel–Haenszel method. The figure was created using Review Manager v. 5.3 software (https://training.cochrane.org).

**Figure 3:**
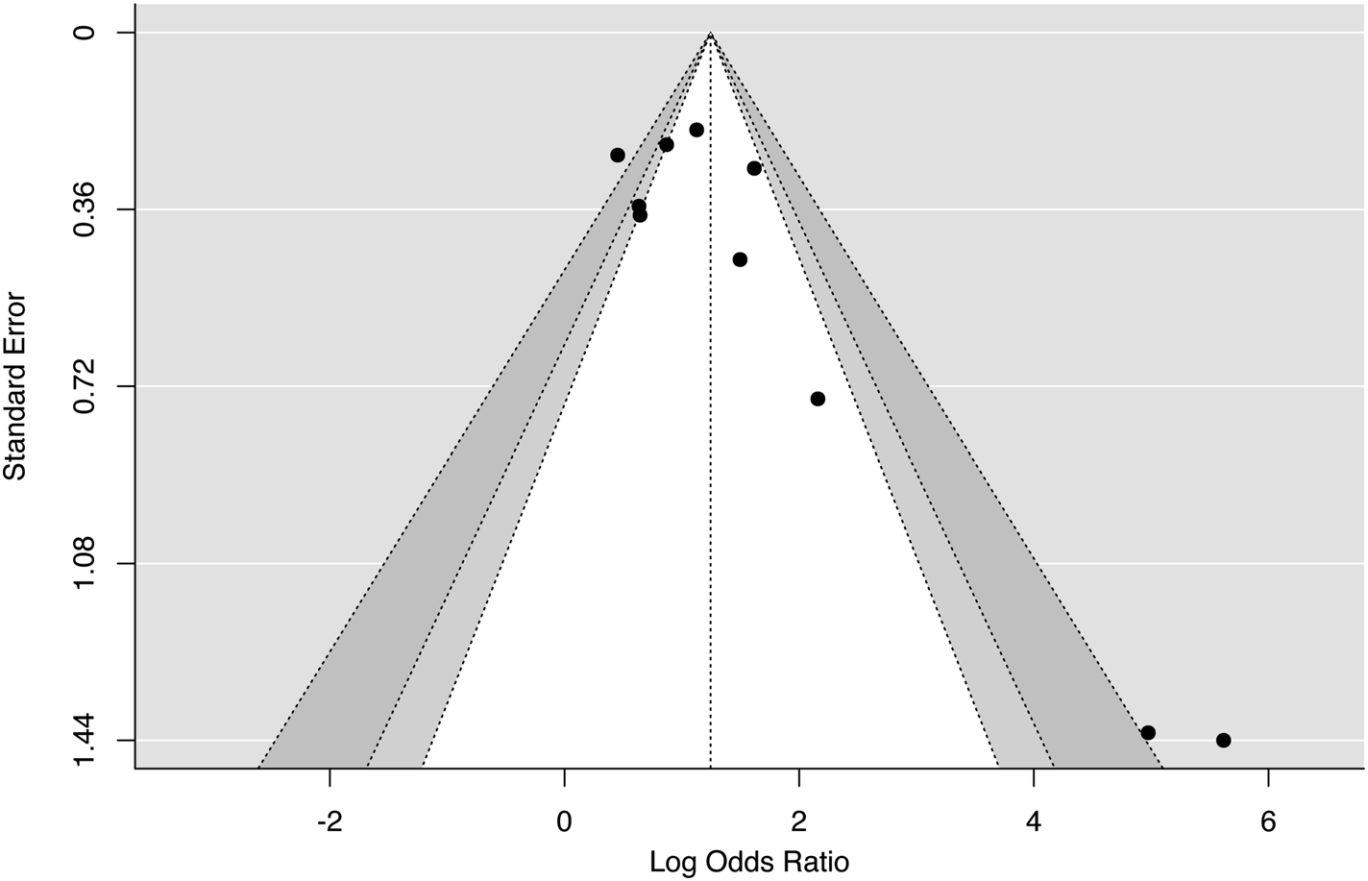
Funnel plot of meta-analysis for ten studies (I^2^ = 77%). The association between chronic exposure to fluoride and cognitive deficit (p < 0.001). The figure was created using Review Manager v. 5.3 software (https://training.cochrane.org).

Ten studies^21,25,28,30,31,34,35,37,38,40^ that provided sufficient data for the analysis were included in the meta-analysis. From the studies selected, it was only possible to run the meta-analysis for IQ, due to the scarcity of investigations on other neurological aspects. People exposed to high F levels accounted for 1383 individuals, and to low levels, 1556 individuals. The results showed an association between high F exposure and decreased IQ (OR 3.88; 95% CI 2.41–6.23; p < 0.00001; I^2^ = 77%), demonstrating a deleterious effect of high levels of F over IQ (Fig. 2). This evidence was qualified as very low (Table 3). It was observed a considerable heterogeneity (I^2^ = 77%, p < 0.00001, Fig. 2) and significant publication bias (p < 0.00001) (Fig. 3).

After performing the sensitivity analysis, three studies were identified as a possible cause of publication bias^25,30,31^, with the detection of a low risk of publication bias (p = 0.25; Figure A, Supplementary material 5) after the exclusion of these studies. However, a considerable heterogeneity was still observed after sensitivity analysis. When the three studies previously identified as possible reason for publication bias were removed from the meta-analysis, the I^2^ index decreased from 77 to 62% (Table B, Supplementary material 5). Therefore, the interpretation of the meta-analysis results after sensitivity analysis is still limited due to the considerable heterogeneity across the studies.

## Discussion

This systematic review and meta-analysis gathered evidence showing that, following the WHO classification of low and high levels in the drinking water, exposure to low/adequate water F levels is not associated with any neurological damage, while exposure to high levels is. The level of evidence for this association, however, was considered very low. Furthermore, the IQ deficit was reported in the marjority of the primary studies identified, and only one article reported others neurological manifestations.

Systematic reviews aim to gather all the available evidence in the literature to answer a guiding question according to predefined eligibility criteria. It uses a well-designed, explicit and systematic methodology to minimize bias, generating reliable results, answers to raised questions and conclusions about certain problems, thus helping in decision making^47,48^. According to the Cochrane systematic reviews manual, this type of study has as main characteristics: clear and well-defined objectives that follow the pre-established eligibility criteria; the methodology must be easily reproducible, well designed and transparent; the survey must be comprehensive, meeting all the necessary eligibility criteria; the included studies must have their results evaluated for validity, assessing the risk of bias; all characteristics of the studies, including their results, must be presented.

Combined with qualitative synthesis, the meta-analysis reunites the quantitative data of the elected studies, thus being able to estimate the effects of the evidence, whether or not it can confirm the individual results of the elected studies of the systematic review^15^. After these qualitative and quantitative analysis, the GRADE tool helps to compile all the obtained results in the systematic review in order to promote an analysis of evidence and its recommendations for an evidence-based practice. This assessment has four levels of recommendations: very low, low, moderate and high.

Despite some variations in the literature on the F concentrations in the drinking water regarded as both effective and safe, it has been often reported that 1 mg/L is the “optimum level”^13,14^ and, as previously mentioned, the concentrations may be adjusted at 0.7–1.2 mg/L, depending on climate, local environment and other sources of F^6^. In line with the above-mentioned observation, the 2017-updated edition of the WHO guidelines for drinking-water quality suggested that F levels must be within the 0.5–1.0 mg/L range in order to promote maximum caries-preventive benefits with minimum risk of dental fluorosis^13,14^. This justifies the threshold set in the present study to dichotomize F exposure into “low” and “high” categories. This is also more relevant from a public health standpoint, given that artificially fluoridated water facilities must comply with the aforementioned levels, whereas higher concentrations are usually related to focal points in areas in which F is naturally present in the water.

The mechanisms by which F can interfere with child neurodevelopment are associated with damage to nervous cells. Evidences suggest that chronic exposure to F in the prenatal and neonatal periods is potentially toxic to the metabolism and physiology of neuronal and glial cells, which leads to changes in processes related to memory and learning^9,49–51^. This is due to the ability of F to cross the placental and blood–brain barriers, especially in developing individuals, who are more susceptible to changes caused by F because they have greater permeability of this barrier and defense mechanisms that are still immature^49,52–54^. In addition, F can influence membrane ion channels, through interaction with the Ras protein, leading to changes in ion flow and nerve cell volume, which can lead to metabolic disturbances, changes in cell function and modification transmission of nerve impulses^49,55^.

According to the WHO, neurological disorders are multifactorial clinical conditions that may be characterized by signs and symptoms with different aspects, as physical functioning limitations, behavioral problems, psychosocial limitations, communicative and cognitive impairments^56^. Among these features, our study focused on cognitive functions due the approach performed by the elected studies. In this sense, it is important to highlight that several techniques, tests and protocols to evaluate the cognitive functions are available^57^, once this central function may be characterized as a complex reunion of processes that aims to classify, recognize and comprise information through reasoning, learning and executing them^58^.

In this context, aiming to evaluate cognitive functions of people exposed to F, the researchers from the elected studies used IQ test varieties as previously mentioned and due to that, different abilities of cognitive functions are evaluated, not having standardized and homogeneous parameters among the tests. Matzel and Sauce^59^ suggested a hierarquical model of intelligence, in which the general ability, i.e., the intelligence is a result from several domains of ability, as reasoning, processing speed, memory and comprehension, which are evaluated by different methodologies. Stanford-Binet IQ method, e.g., includes tests of different abilities, which estimate the intelligence after and aggregate performance across the tests. While, the Raven's Standard Progressive Matrices is based on a unique ability and in the test, the main feature is that there is an increase on the difficult of perceptual reasoning^60^.

The studies included individuals with ages ranging from 6 to 18 years of age. From epidemiological point of view, this is not interesting, because intelligence tests were applied to participants with very different degrees of neurodevelopment. Data extraction indicates that all eligible studies were concentrated in the Asian continent. These data reflect the remarkable influence of the geographical aspect on the epidemiology of clinical manifestations resulting from F exposure. The availability of naturally occurring high concentration fluoridated compounds in drinking water used by rural communities increases their susceptibility to the adverse effects of F. Considering this aspect, a systematic review proposed to evaluate the neurotoxic effects of F from studies conducted specifically in the Chinese territory^9^, due to the high number of publications on this subject that sometimes has restricted dissemination due to language barrier.

The methodological quality analyses of the studies detected serious problems related to the quality of sample, measurements and outcomes. There were also problems related to the absence of randomization, sample size calculation and blinding, which increase the risk of bias and limit the inference capacity of studies on the neurotoxic effects of F.

Most studies did not assess the individual level of exposure to F, i.e., by urinary F samples. The F concentration in drinking water in regions with high and low F levels was the most reported method. However, there were also studies that used secondary data or did not report the F content in water, which significantly compromises the findings of these investigations. Furthermore, it should be considered that some studies used creatinine-adjusted urinary F concentrations to account for urinary dilution which may cause an additional bias^61^, since renal dysfunction in children may be associated with neurocognitive impairments^62^.

Another point worth mentioning is the increased risk of water contamination by other substances in the areas of naturally occurring F. Although some authors consider it unlikely that the effects attributed to F neurotoxicity can be triggered by other contaminants^9^, it is possible that the absence of control in relation to these parameters generates confounding factors. To ensure the balance of electrical charges, water with higher concentrations of endemically occurring F must contain higher concentrations of positive ions to balance out the F. This may affect the pH of the water or result in greater contamination by electropositive water contaminants, for example aluminum, zinc, arsenic, lead, mercury, and other metals and metalloids^61^.

Following the parameters of GRADE, the level of evidence was considered as very low even for individuals exposed to high doses of F, due to imprecision problems (Table 3). This result is related to the types of studies included in this systematic review, as the level of evidence in observational studies starts at a very low level, which can only increase if the study meets the other criteria of this evaluation. Despite the large numbers of participants in the analysis, detected problems of inaccuracy can be elucidated by possible methodological disparities in the studies that might interfere in the intelligence quotient (IQ) analysis and neurological manifestations.

Another important limitation to be considered is the predominance of cross-sectional studies in this systematic review. Cross-sectional and ecological studies do not allow the establishment of cause-and-effect relationships. They are useful for investigating the effect of environmental exposures related to acute processes, as the time interval between exposure and measurement of physiological parameters is close. Therefore, cross-sectional studies are not the ideal model to assess the effect of chronic F exposure on a parameter such as human intelligence^61^. Longitudinal studies, on the other hand, are considered the most appropriate to assess chronic conditions, as by allowing the long-term follow-up of individuals, they make it possible to infer causality^63^.

To sum up, despite the elected studies showed an association between F exposure and IQ deficit, this association was only observed for individuals exposed to levels above those regarded as safe, and the evidence certainty for this association is very low. Within the above-mentioned limitations, the results of the present systematic review demonstrated that exposure to fluoridated water at levels recommended by the WHO can be considered as safe, as it is not associated with IQ impairment.

## Conclusion

Although the findings of this meta-analysis indicated that IQ damage can be triggered only by exposure to F at levels that exceed those recommended as a public health measure, the high heterogeneity observed compromise the final conclusions obtained by quantitative analyses. Thus, based on the evidence available on the topic, it is not possible to state neither any association or the lack of an association between F exposure and any neurological disorder.

## Supplementary Information

Supplementary Information 1.

Supplementary Information 2.

Supplementary Information 3.

Supplementary Information 4.

Supplementary Information 5.

## Data Availability

All the data is available within the article and on the supplementary materials.

## Abbreviations

F: Fluoride
WHO: World Health Organization
IQ: Intelligence Quotient
PRISMA: Preferred reporting items for systematic reviews and meta-analyses
NA: Not Applicable
CI: Confidence Interval
GRADE: Grading of Recommendations Assessment, Development and Evaluation
WPPSI: Wechsler Preschool Guidelines and Primary Intelligence Scale
