# Supplementary material for: A systematic review and meta-analysis of the association between fluoride exposure and neurological disorders
Source: Sci Rep. 2021 Nov 22;11:22659. doi: 10.1038/s41598-021-99688-w (PMC8609002; doi:10.1038/s41598-021-99688-w)
Supplement: Supplementary file 1 — Supplementary Information 1. [file 41598_2021_99688_MOESM1_ESM.docx]

**Appendix Table 1.** Search strategies in the different databases.

| BATABASES | KEYWORDS |
| --- | --- |
| PUBMED | #1 AND #2 |
|  | #1 (((((Fluorine[MeSH Terms] OR Fluorine[Title/Abstract] OR Fluorine-19[Title/Abstract] OR Fluorine 19[Title/Abstract] OR Fluorides[MeSH Terms] OR Fluorides[Title/Abstract] OR Fluoride[Title/Abstract] OR Fluorine Compounds[MeSH Terms] OR Fluorine Compounds[Title/Abstract] OR Compounds, Fluorine[Title/Abstract] OR Fluoride Poisoning[MeSH Terms] OR Fluoride Poisoning[Title/Abstract] OR Poisoning, Fluoride[Title/Abstract] OR Fluoride Poisonings[Title/Abstract] OR Poisonings, Fluoride[Title/Abstract])) |
|  | #2   (((Neurobehavioral manifestations[MeSH Terms] OR Neurobehavioral manifestations[Title/Abstract] OR Manifestation, Neurobehavioral[Title/Abstract] OR Manifestations, Neurobehavioral[Title/Abstract] OR Neurobehavioral Manifestation[Title/Abstract] OR Cognitive Symptoms[Title/Abstract] OR Cognitive Symptom[Title/Abstract] OR Symptom, Cognitive[Title/Abstract] OR Symptoms, Cognitive[Title/Abstract] OR Signs Symptoms, Neurobehavioral[Title/Abstract] OR Cognitive Manifestations[Title/Abstract] OR Cognitive Manifestation[Title/Abstract] OR Manifestation, Cognitive[Title/Abstract] OR Manifestations, Cognitive[Title/Abstract] OR Neurobehavioral Signs Symptoms[Title/Abstract] OR Nervous System Disease[MeSH Terms] OR Nervous System Disease[Title/Abstract] OR Disease, Nervous System[Title/Abstract] OR Diseases, Nervous System[Title/Abstract] OR Nervous System Disease[Title/Abstract] OR Neurologic Disorders[Title/Abstract] OR Disorder, Neurologic[Title/Abstract] OR Disorders, Neurologic[Title/Abstract] OR Neurologic Disorder[Title/Abstract] OR Neurological Disorders[Title/Abstract] OR Disorder, Neurological[Title/Abstract] OR Disorders, Neurological[Title/Abstract] OR Neurological Disorder[Title/Abstract] OR Nervous System Disorders[Title/Abstract] OR Disorder, Nervous System[Title/Abstract] OR Disorders, Nervous System[Title/Abstract] OR Nervous System Disorder[Title/Abstract] OR Neurologic manifestations[MeSH Terms] OR Neurologic manifestations[Title/Abstract] OR Manifestation, Neurologic[Title/Abstract] OR Neurological Manifestations[Title/Abstract] OR Neurologic Manifestation[Title/Abstract] OR Neurologic Signs Symptoms[Title/Abstract] OR Manifestations, Neurologic[Title/Abstract] OR Manifestations, Neurological[Title/Abstract] OR Manifestation, Neurological[Title/Abstract] OR Neurological Manifestation[Title/Abstract] OR Neurologic Deficits[Title/Abstract] OR Deficit, Neurologic[Title/Abstract] OR Deficits, Neurologic[Title/Abstract] OR Neurologic Deficit[Title/Abstract] OR Neurologic Symptoms[Title/Abstract] OR Neurologic Symptom[Title/Abstract] OR Symptom, Neurologic[Title/Abstract] OR Symptoms, Neurologic[Title/Abstract] OR Neurologic Findings[Title/Abstract] OR Finding, Neurologic[Title/Abstract] OR Findings, Neurologic[Title/Abstract] OR Neurologic Finding[Title/Abstract] OR Neurologic Signs[Title/Abstract] OR Neurologic Sign[Title/Abstract] OR Sign, Neurologic[Title/Abstract] OR Signs, Neurologic[Title/Abstract] OR Focal Neurologic Deficits[Title/Abstract] OR Deficit, Focal Neurologic[Title/Abstract] OR Deficits, Focal Neurologic[Title/Abstract] OR Focal Neurologic Deficit[Title/Abstract] OR Neurologic Deficit, Focal[Title/Abstract] OR Neurologic Deficits, Focal[Title/Abstract] OR Neurologic Dysfunction[Title/Abstract] OR Dysfunction, Neurologic[Title/Abstract] OR Dysfunctions, Neurologic[Title/Abstract] OR Neurologic Dysfunctions[Title/Abstract] OR Intelligence[MeSH Terms] OR Intelligence[Title/Abstract] OR Intelligence Quotient[Title/Abstract])))))) |
| SCOPUS | #1 AND #2 |
|  | #1 ( ( TITLE-ABS-KEY ( fluorine ) OR TITLE-ABS-KEY ( fluorine-19 ) OR TITLE-ABS-KEY ( fluorine 19 ) OR TITLE-ABS-KEY ( fluorides ) OR TITLE-ABS-KEY ( fluoride ) OR TITLE-ABS-KEY ( fluorine AND compounds ) OR TITLE-ABS-KEY ( compounds, AND fluorine ) OR TITLE-ABS-KEY ( fluoride AND poisoning ) OR TITLE-ABS-KEY ( poisoning, AND fluoride ) OR TITLE-ABS-KEY ( fluoride AND poisonings ) OR TITLE-ABS-KEY ( poisonings, AND fluoride ) ) ) |
|  | #2 ( ( TITLE-ABS-KEY ( neurobehavioral AND manifestations ) OR TITLE-ABS-KEY ( manifestation, AND neurobehavioral ) OR TITLE-ABS-KEY ( manifestations, AND neurobehavioral ) OR TITLE-ABS-KEY ( neurobehavioral AND manifestation ) OR TITLE-ABS-KEY ( cognitive AND symptoms ) OR TITLE-ABS-KEY ( cognitive AND symptom ) OR TITLE-ABS-KEY ( symptom, AND cognitive ) OR TITLE-ABS-KEY ( symptoms, AND cognitive ) OR TITLE-ABS-KEY ( signs AND symptoms, AND neurobehavioral ) OR TITLE-ABS-KEY ( cognitive AND manifestations ) OR TITLE-ABS-KEY ( cognitive AND manifestation ) OR TITLE-ABS-KEY ( manifestation, AND cognitive ) OR TITLE-ABS-KEY ( manifestations, AND cognitive ) OR TITLE-ABS-KEY ( neurobehavioral AND signs AND symptoms ) ) OR ( ( TITLE-ABS-KEY ( nervous AND system AND disease ) OR TITLE-ABS-KEY ( disease, AND nervous AND system ) OR TITLE-ABS-KEY ( diseases, AND nervous AND system ) OR TITLE-ABS-KEY ( nervous AND system AND disease ) OR TITLE-ABS-KEY ( neurologic AND disorders ) OR TITLE-ABS-KEY ( disorder, AND neurologic ) OR TITLE-ABS-KEY ( disorders, AND neurologic ) OR TITLE-ABS-KEY ( neurologic AND disorder ) OR TITLE-ABS-KEY ( neurological AND disorders ) OR TITLE-ABS-KEY ( disorder, AND neurological ) OR TITLE-ABS-KEY ( disorders, AND neurological ) OR TITLE-ABS-KEY ( neurological AND disorder ) OR TITLE-ABS-KEY ( nervous AND system AND disorders ) OR TITLE-ABS-KEY ( disorder, AND nervous AND system ) OR TITLE-ABS-KEY ( disorders, AND nervous AND system ) OR TITLE-ABS-KEY ( nervous AND system AND disorder ) ) ) OR ( ( TITLE-ABS-KEY ( neurologic AND manifestations ) OR TITLE-ABS-KEY ( manifestation, AND neurologic ) OR TITLE-ABS-KEY ( neurological AND manifestations ) OR TITLE-ABS-KEY ( neurologic AND manifestation ) OR TITLE-ABS-KEY ( neurologic AND signs AND symptoms ) OR TITLE-ABS-KEY ( manifestations, AND neurologic ) OR TITLE-ABS-KEY ( manifestations, AND neurological ) OR TITLE-ABS-KEY ( manifestation, AND neurological ) OR TITLE-ABS-KEY ( neurological AND manifestation ) OR TITLE-ABS-KEY ( neurologic AND deficits ) OR TITLE-ABS-KEY ( deficit, AND neurologic ) OR TITLE-ABS-KEY ( deficits, AND neurologic ) OR TITLE-ABS-KEY ( neurologic AND deficit ) OR TITLE-ABS-KEY ( neurologic AND symptoms ) OR TITLE-ABS-KEY ( neurologic AND symptom ) OR TITLE-ABS-KEY ( symptom, AND neurologic ) OR TITLE-ABS-KEY ( symptoms, AND neurologic ) OR TITLE-ABS-KEY ( neurologic AND findings ) OR TITLE-ABS-KEY ( finding, AND neurologic ) OR TITLE-ABS-KEY ( findings, AND neurologic ) OR TITLE-ABS-KEY ( neurologic AND finding ) OR TITLE-ABS-KEY ( neurologic AND signs ) ) ) OR ( ( TITLE-ABS-KEY ( neurologic AND sign ) OR TITLE-ABS-KEY ( sign, AND neurologic ) OR TITLE-ABS-KEY ( signs, AND neurologic ) OR TITLE-ABS-KEY ( focal AND neurologic AND deficits ) OR TITLE-ABS-KEY ( deficit, AND focal AND neurologic ) OR TITLE-ABS-KEY ( deficits, AND focal AND neurologic ) OR TITLE-ABS-KEY ( focal AND neurologic AND deficit ) OR TITLE-ABS-KEY ( neurologic AND deficit, AND focal ) OR TITLE-ABS-KEY ( neurologic AND deficits, AND focal ) OR TITLE-ABS-KEY ( neurologic AND dysfunction ) OR TITLE-ABS-KEY ( dysfunction, AND neurologic ) OR TITLE-ABS-KEY ( dysfunctions, AND neurologic ) OR TITLE-ABS-KEY ( neurologic AND dysfunctions ) OR TITLE-ABS-KEY ( intelligence ) OR TITLE-ABS-KEY ( intelligence AND quotient ) ) ) ) |
| WEB OF SCIENCE | #1 AND #2 |
|  | #1 Tópico: (Fluorine) OR Tópico: (Fluorine-19) OR Tópico: (Fluorine 19) OR Tópico: (Fluorides) OR Tópico: (Fluoride) OR Tópico: (Fluorine Compounds) OR Tópico: (Compounds, Fluorine) OR Tópico: (Fluoride Poisoning) OR Tópico: (Poisoning, Fluoride) OR Tópico: (Fluoride Poisonings) OR Tópico: (Poisonings, Fluoride) |
|  | #2 Tópico: (Neurobehavioral manifestations) OR Tópico: (Manifestation, Neurobehavioral) OR Tópico: (Manifestations, Neurobehavioral) OR Tópico: (Neurobehavioral Manifestation) OR Tópico: (Cognitive Symptoms) OR Tópico: (Cognitive Symptom) OR Tópico: (Symptom, Cognitive) OR Tópico: (Symptoms, Cognitive) OR Tópico: (Signs Symptoms, Neurobehavioral) OR Tópico: (Cognitive Manifestations) OR Tópico: (Cognitive Manifestation) OR Tópico: (Manifestation, Cognitive) OR Tópico: (Manifestations, Cognitive) OR Tópico: (Neurobehavioral Signs Symptoms) OR Tópico: (Nervous System Disease) OR Tópico: (Disease, Nervous System) OR Tópico: (Diseases, Nervous System) OR Tópico: (Nervous System Disease) OR Tópico: (Neurologic Disorders) OR Tópico: (Disorder, Neurologic) OR Tópico: (Disorders, Neurologic) OR Tópico: (Neurologic Disorder) OR Tópico: (Neurological Disorders) OR Tópico: (Disorder, Neurological) OR Tópico: (Disorders, Neurological) OR Tópico: (Neurological Disorder) OR Tópico: (Nervous System Disorders) OR Tópico: (Disorder, Nervous System) OR Tópico: (Disorders, Nervous System) OR Tópico: (Nervous System Disorder) OR Tópico: (Neurologic manifestations) OR Tópico: (Manifestation, Neurologic) OR Tópico: (Neurological Manifestations) OR Tópico: (Neurologic Manifestation) OR Tópico: (Neurologic Signs Symptoms) OR Tópico: (Manifestations, Neurologic) OR Tópico: (Manifestations, Neurological) OR Tópico: (Manifestation, Neurological) OR Tópico: (Neurological Manifestation) OR Tópico: (Neurologic Deficits) OR Tópico: (Deficit, Neurologic) OR Tópico: (Deficits, Neurologic) OR Tópico: (Neurologic Deficit) OR Tópico: (Neurologic Symptoms) OR Tópico: (Neurologic Symptom) OR Tópico: (Symptom, Neurologic) OR Tópico: (Symptoms, Neurologic) OR Tópico: (Neurologic Findings) OR Tópico: (Finding, Neurologic) OR Tópico: (Findings, Neurologic) OR Tópico: (Neurologic Finding) OR Tópico: (Neurologic Signs) OR Tópico: (Neurologic Sign) OR Tópico: (Sign, Neurologic) OR Tópico: (Signs, Neurologic) OR Tópico: (Focal Neurologic Deficits) OR Tópico: (Deficit, Focal Neurologic) OR Tópico: (Deficits, Focal Neurologic) OR Tópico: (Focal Neurologic Deficit) OR Tópico: (Neurologic Deficit, Focal) OR Tópico: (Neurologic Deficits, Focal) OR Tópico: (Neurologic Dysfunction) OR Tópico: (Dysfunction, Neurologic) OR Tópico: (Dysfunctions, Neurologic) OR Tópico: (Neurologic Dysfunctions) OR Tópico: (Intelligence) OR Tópico: (Intelligence Quotient) |
|  | #1 AND #2 |
| COCHRANE | #1 Fluorine:ti,ab,kw or Fluorine-19:ti,ab,kw or Fluorine 19:ti,ab,kw or Fluorides:ti,ab,kw or Fluoride:ti,ab,kw or Fluorine Compounds:ti,ab,kw or Compounds, Fluorine:ti,ab,kw or Fluoride Poisoning:ti,ab,kw or Poisoning, Fluoride:ti,ab,kw or Fluoride Poisonings:ti,ab,kw or Poisonings, Fluoride:ti,ab,kw |
|  | #2 Neurobehavioral manifestations:ti,ab,kw or Manifestation, Neurobehavioral:ti,ab,kw or Manifestations, Neurobehavioral:ti,ab,kw or Neurobehavioral Manifestation:ti,ab,kw or Cognitive Symptoms:ti,ab,kw or Cognitive Symptom:ti,ab,kw or Symptom, Cognitive:ti,ab,kw or Symptoms, Cognitive:ti,ab,kw or Signs Symptoms, Neurobehavioral:ti,ab,kw or Cognitive Manifestations:ti,ab,kw or Cognitive Manifestation:ti,ab,kw or Manifestation, Cognitive:ti,ab,kw or Manifestations, Cognitive:ti,ab,kw or Neurobehavioral Signs Symptoms:ti,ab,kw or Nervous System Disease:ti,ab,kw or Disease, Nervous System:ti,ab,kw or Diseases, Nervous System:ti,ab,kw or Nervous System Disease:ti,ab,kw or Neurologic Disorders:ti,ab,kw or Disorder, Neurologic:ti,ab,kw or Disorders, Neurologic:ti,ab,kw or Neurologic Disorder:ti,ab,kw or Neurological Disorders:ti,ab,kw or Disorder, Neurological:ti,ab,kw or Disorders, Neurological:ti,ab,kw or Neurological Disorder:ti,ab,kw or Nervous System Disorders:ti,ab,kw or Disorder, Nervous System:ti,ab,kw or Disorders, Nervous System:ti,ab,kw or Nervous System Disorder:ti,ab,kw or Neurologic manifestations:ti,ab,kw or Manifestation, Neurologic:ti,ab,kw or Neurological Manifestations:ti,ab,kw or Neurologic Manifestation:ti,ab,kw or Neurologic Signs Symptoms:ti,ab,kw or Manifestations, Neurologic:ti,ab,kw or Manifestations, Neurological:ti,ab,kw or Manifestation, Neurological:ti,ab,kw or Neurological Manifestation:ti,ab,kw or Neurologic Deficits:ti,ab,kw or Deficit, Neurologic:ti,ab,kw or Deficits, Neurologic:ti,ab,kw or Neurologic Deficit:ti,ab,kw or Neurologic Symptoms:ti,ab,kw or Neurologic Symptom:ti,ab,kw or Symptom, Neurologic:ti,ab,kw or Symptoms, Neurologic:ti,ab,kw or Neurologic Findings:ti,ab,kw or Finding, Neurologic:ti,ab,kw or Findings, Neurologic:ti,ab,kw or Neurologic Finding:ti,ab,kw or Neurologic Signs:ti,ab,kw or Neurologic Sign:ti,ab,kw or Sign, Neurologic:ti,ab,kw or Signs, Neurologic:ti,ab,kw or Focal Neurologic Deficits:ti,ab,kw or Deficit, Focal Neurologic:ti,ab,kw or Deficits, Focal Neurologic:ti,ab,kw or Focal Neurologic Deficit:ti,ab,kw or Neurologic Deficit, Focal:ti,ab,kw or Neurologic Deficits, Focal:ti,ab,kw or Neurologic Dysfunction:ti,ab,kw or Dysfunction, Neurologic:ti,ab,kw or Dysfunctions, Neurologic:ti,ab,kw or Neurologic Dysfunctions:ti,ab,kw or Intelligence:ti,ab,kw or Intelligence Quotient:ti,ab,kw |
|  | #1 AND #2 |
| LILACS | 1# (Fluorine) or (Fluorine-19) or (Fluorine 19) or (Fluorides) or (Fluoride) or (Fluorine Compounds) or (Compounds, Fluorine) or (Fluoride Poisoning) or (Poisoning, Fluoride) or (Fluoride Poisonings) or (Poisonings, Fluoride) |
|  | 2# (Neurobehavioral manifestations) or (Manifestation, Neurobehavioral) or (Manifestations, Neurobehavioral) or (Neurobehavioral Manifestation) or (Cognitive Symptoms) or (Cognitive Symptom) or (Symptom, Cognitive) or (Symptoms, Cognitive) or (Signs Symptoms, Neurobehavioral) or (Cognitive Manifestations) or (Cognitive Manifestation) or (Manifestation, Cognitive) or (Manifestations, Cognitive) or (Neurobehavioral Signs Symptoms) or (Nervous System Disease) or (Disease, Nervous System) or (Diseases, Nervous System) or (Nervous System Disease) or (Neurologic Disorders) or (Disorder, Neurologic) or (Disorders, Neurologic) or (Neurologic Disorder) or (Neurological Disorders) or (Disorder, Neurological) or (Disorders, Neurological) or (Neurological Disorder) or (Nervous System Disorders) or (Disorder, Nervous System) or (Disorders, Nervous System) or (Nervous System Disorder) or (Neurologic manifestations) or (Manifestation, Neurologic) or (Neurological Manifestations) or (Neurologic Manifestation) or (Neurologic Signs Symptoms) or (Manifestations, Neurologic) or (Manifestations, Neurological) or (Manifestation, Neurological) or (Neurological Manifestation) or (Neurologic Deficits) or (Deficit, Neurologic) or (Deficits, Neurologic) or (Neurologic Deficit) or (Neurologic Symptoms) or (Neurologic Symptom) or (Symptom, Neurologic) or (Symptoms, Neurologic) or (Neurologic Findings) or (Finding, Neurologic) or (Findings, Neurologic) or (Neurologic Finding) or (Neurologic Signs) or (Neurologic Sign) or (Sign, Neurologic) or (Signs, Neurologic) or (Focal Neurologic Deficits) or (Deficit, Focal Neurologic) or (Deficits, Focal Neurologic) or (Focal Neurologic Deficit) or (Neurologic Deficit, Focal) or (Neurologic Deficits, Focal) or (Neurologic Dysfunction) or (Dysfunction, Neurologic) or (Dysfunctions, Neurologic) or (Neurologic Dysfunctions) or (Intelligence) or (Intelligence Quotient) |
|  | #1 AND #2 |
| GOOGLE SCHOLAR | Fluoride+"Neurological manifestations"+Intelligence |
