# Supplementary material for: A systematic review and meta-analysis of the association between fluoride exposure and neurological disorders
Source: Sci Rep. 2021 Nov 22;11:22659. doi: 10.1038/s41598-021-99688-w (PMC8609002; doi:10.1038/s41598-021-99688-w)
Supplement: Supplementary file 2 — Supplementary Information 2. [file 41598_2021_99688_MOESM2_ESM.docx]

**Appendix Table 2.** Domains and Risk of Bias considered in Risk of Bias evaluation according to Fowkes and Fulton [10].

| **Guideline** | **Checklist** | **Description** |
| --- | --- | --- |
| Study design apropriate to objectives? | objective common design | It was assigned (0) for studies whose design was compatible with the proposal and (++) for studies with inappropriate design for the purposes. |
|  | prevalence Cross-sectional |  |
|  | Prognosis Cohort |  |
|  | Treatment Controled trial |  |
|  | Cause Cohort, case-control, cross-sectional |  |
| Study sample representative? | Source of sample | (0) was considered in cases of detailed sample origin, (+) when specifying the origin of only one group and (++) when there was no specification as to the origin of the groups. |
|  | Sampling method | (0) was attributed to the occurrence of randomization, (+) to the randomization of one of the groups and (++) to the absence of randomization of the groups. |
|  | Sample size | It was considered a minor problem (+) when the representativeness of the sample was not clear in the study or the sample calculation was not reported. For a larger problem (++) the absence of sample calculation was considered and the number of participants was less than 50 participants and (0) for the absence of the factors mentioned above. |
|  | Entry criteria/exclusion | A minor problem (+) was attributed when the control group and case reported the presence of elderly participants (> 65 years), coexposure to substances other than fluorine and additional consumption of fluoride by supplements, toothpastes, mouthwashes. In the case of more than one item mentioned above, it was considered a major problem (++). |
|  | Non-respondents | It was considered (0) when all recruited individuals participated in the study, (+) for the occurrence of refusal, but without compromising the sample and (++) for when there was refusal and compromising the sample size. |
| Control group acceptable? | Definition of controls | It was assigned (0) when all the characteristics of the control group were described, (+) when some information was pending such as, the origin of the control group, the group selection criteria, the fluoride concentrations to which they are exposed and (+ +) for when two or more items were pending. |
|  | Source of controls | It was considered (0) when the control group was mentioned and (++) when the origin of the control group was not mentioned. |
|  | Matching/randomization | In this item, (0) was applied when there was pairing between groups, (+) when there was no description regarding randomization, however, groups were paired and (++) when there was no description regarding randomization and it was not done pairing of groups. |
|  | Comparable characteristics | It was assigned (0) for paired or unpaired groups due to the impossibility of being adjusted later and (++) for the presence of variables that were neither paired nor adjusted. |
| Quality of measurements and outcomes? | Validity | It was considered (0) when the applied cognitive capacity assessment method was adequate, (+) when the assessment method was questionable or not validated, but with adequate specificity and good sensitivity and (++) when a method was used not validated, and it did not have adequate specificity or good sensitivity. |
|  | Reproducibility | It was considered (0) when the evaluation methods were well described, capable of being reproduced; (+) when there was impairment of the neurological assessment, such as the absence of a description of the assessment of neurological changes, assessments carried out at different times, application of different methods for assessing the groups, and (++) when two or more of the previous items were present. |
|  | Blindness | It was considered if the condition of the study participants was the sign “blind” (0), in cases of “not blind” the sign (++). |
|  | Quality control | They were considered a problem when the evaluators were not calibrated; the statistical test was not reported; application of neurological assessment tests were performed by untrained professionals; fluoride concentrations were based on information available from other sources; there was no estimation of fluoride concentrations at an environmental and / or individual level or the references used were not mentioned. When one of these characteristics were present, it was considered as a minor problem (+) and a major problem (++) if more than one of these characteristics were present. |
| Completeness | Compliance | It was assigned (0) when the sample size remained from the beginning to the end or decreases without compromising the power of the test; (+) when there was a difference in the sample size at the end of the study, compromising the power of the test, however there was justification and adjustment and (++) when there was a difference in the sample size at the end of the study, compromising the power of the test, without justification and adjust. |
|  | Drop outs | The sign (0) was when assigned, there were no losses during the study; (+) when there was a dropout that compromised the inclusion criteria, such as age, sex and (++) when there was a dropout and compromised more than one criterion. |
|  | Deaths | This item was assessed as “Not Applicable” (NA), due to the type of PECO strategy. |
|  | Missing data | The sign (0) was considered when there was no data loss; (+) when there was loss of sample data without compromising the final statistical analysis and (++) when there was loss of sample data compromising the final statistical analysis. |
| Distorting influences? | Extraneous treatments | It was considered (0) when there were no external influences; (+) when there were external influences, but without interference in the results and (++) when there were external influences with interference in the results. |
|  | Contamination | This item was assessed as “Not Applicable” (NA), due to the type of PECO strategy. |
|  | Changes over time | It was assigned (0) when the data were collected in the same period of time; (+) for data collected from the control group and the study group at different times, with distortions occurring and (++) for the previous item associated with data from studies already published. |
|  | Confounding factors | The problems considered were men and women over the age of 65; comparison between regions with different demographic characteristics and standard of living; use of additional fluoride supplementation in the diet; presence of neurodegenerative diseases. A "minor" problem (+) was assigned when 1 or 2 of these characteristics were present and a "major" problem (++) if there were 3 or more. |
|  | Distortion reduced by analysis | It was considered (0) when there was citation of the adjustments of the covariates that presented distortions; (+) when the adjustment was mentioned, but without clarifying the criteria and (++) when there were covariates with distortions and there was no adjustment. |
| Summary questions | Bias: Are the results erroneously biased in certain direction? | YES or "NO" answers were assigned for each question. If the answer is NO at the three questions, the article is considered reliable, with low risk of bias. |
|  | Confounding: Are there any serious confusing or other distoring influences? |  |
|  | Chance: Is it likely that the results ocurred by chance? |  |
