# Supplementary material for: A systematic review and meta-analysis of the association between fluoride exposure and neurological disorders
Source: Sci Rep. 2021 Nov 22;11:22659. doi: 10.1038/s41598-021-99688-w (PMC8609002; doi:10.1038/s41598-021-99688-w)
Supplement: Supplementary file 3 — Supplementary Information 3. [file 41598_2021_99688_MOESM3_ESM.docx]

| **Table 2.** List of excluded studies with reason. | | |
| --- | --- | --- |
| **Reference** | **Title** | **Reason for exclusion** |
| Bai *et al*. (2014) | Intelligence and growth development of children in coal-burning-borne arsenism and fluorosis areas: An investigation study | Coexposure |
| Barberio *et al*. (2017) | Fluoride exposure and reported learning disability diagnosis among Canadian children: Implications for community water fluoridation | Absence of IQ comparison considering high and low fluoride exposure regions |
| Broadbent *et al*. (2015) | Community Water Fluoridation and Intelligence: Prospective Study in New Zealand | Absence of IQ comparison considering high and low fluoride exposure regions |
| Das *et al*. (2016) | Dental fluorosis and urinary fluoride concentration as a reflection of fluoride exposure and its impact on IQ level and BMI of children of Laxmisagar, Simlapal Block of Bankura District, W.B., India | Absence of IQ comparison considering high and low fluoride exposure regions |
| Ding *et al*. (2011) | The relationships between low levels of urine fluoride on children's intelligence, dental fluorosis in endemic fluorosis areas in Hulunbuir, Inner Mongolia, China | Absence of IQ comparison considering high and low fluoride exposure regions |
| He *et al*. (2010) | Investigation of children's intelligence quotient and dental fluorosis in drinking water-type of endemic fluorosis area in Pucheng county Shaanxi province before and after drinking water change | Absence of IQ comparison considering high and low fluoride exposure regions |
| Li *et al*. (1995) | Effect of fluoride exposure on intelligence in children | Absence of IQ comparison considering high and low fluoride exposure regions |
| Li *et al*. (2003) | Effects of endemic fluoride poisoning on the intellectual development of children in Baotou | Absence of IQ comparison considering high and low fluoride exposure regions |
| Liu *et al*. (2000) | Report on the intellectual ability of children living in high-fluoride water areas | Duplicate sample |
| Lou *et al*. (2020) | Refinement Impairments of Verbal-Performance Intelligent Quotient in Children Exposed to Fluoride Produced by Coal Burning | Absence of IQ comparison considering high and low fluoride exposure regions |
| Mustafa *et al*. (2018) | The relationship between the fluoride levels in drinking water and the schooling performance of children in rural areas of Khartoum State, Sudan | Absence of IQ comparison considering high and low fluoride exposure regions |
| Ren *et al*. (1989) | A study of the intellectual ability of 8-14 year-old children in high fluoride, low iodine areas | Coexposure |
| Rocha-Amador *et al*. (2007) | Decreased intelligence in children and exposure to fluoride and arsenic in drinking water | Coexposure |
| Sharma *et al*. (2016) | Effect of fluoride exposure through drinking water on the oral health status and intelligence profile of school children of district Una, Himachal Pradesh: an interim analysis | Absence of IQ comparison considering high and low fluoride exposure regions |
| Soto-Barreras *et al*. (2019) | Effect of fluoride in drinking water on dental caries and IQ in children | Absence of IQ comparison considering high and low fluoride exposure regions |
| Till *et al*. (2020) | Fluoride exposure from infant formula and child IQ in a Canadian birth cohort | Absence of IQ comparison considering high and low fluoride exposure regions |
| Wang *et al*. (2020) | Thyroid function, intelligence, and low-moderate fluoride exposure among Chinese school-age children | Absence of IQ comparison considering high and low fluoride exposure regions |
| Wang *et al*. (2005) | Investigation and evaluation on intelligence and growth of children in endemic fluorosis and arsenism areas | Duplicate sample |
| Wang *et al*. (2005) | The effects of endemic fluoride poisoning caused by coal burning on the physical development and intelligence of children | Absence of IQ comparison considering high and low fluoride exposure regions |
| Wei *et al*. (2014) | The effects of comprehensive control measures on intelligence of school-age children in coal-burning-borne endemic fluorosis areas | Absence of IQ comparison considering high and low fluoride exposure regions |
| Xiang *et al*. (2007) | Serum fluoride level and children's intelligence quotient in two villages in China | Duplicate sample |
| Xiang *et al*. (2011) | Analysis of children's serum fluoride levels in relation to intelligence scores in a high and low fluoride water village in China | Duplicate sample |
| Yang *et al*. (1994) | Effects of high iodine and high fluorine on children's intelligence and the metabolism of iodine and fluorine | Coexposure |
