# Supplementary material for: A systematic review and meta-analysis of the association between fluoride exposure and neurological disorders
Source: Sci Rep. 2021 Nov 22;11:22659. doi: 10.1038/s41598-021-99688-w (PMC8609002; doi:10.1038/s41598-021-99688-w)
Supplement: Supplementary file 5 — Supplementary Information 5. [file 41598_2021_99688_MOESM5_ESM.docx]

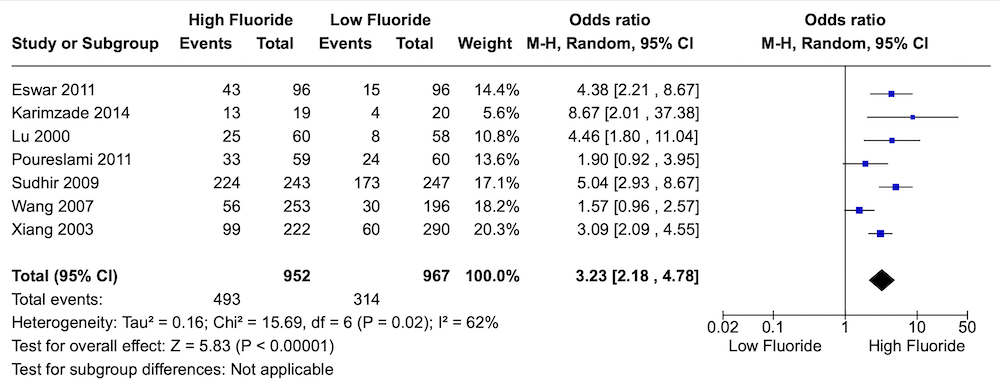


Figure A: Funnel plot after sensitivity analysis (p=0.25 after Egger’s test).


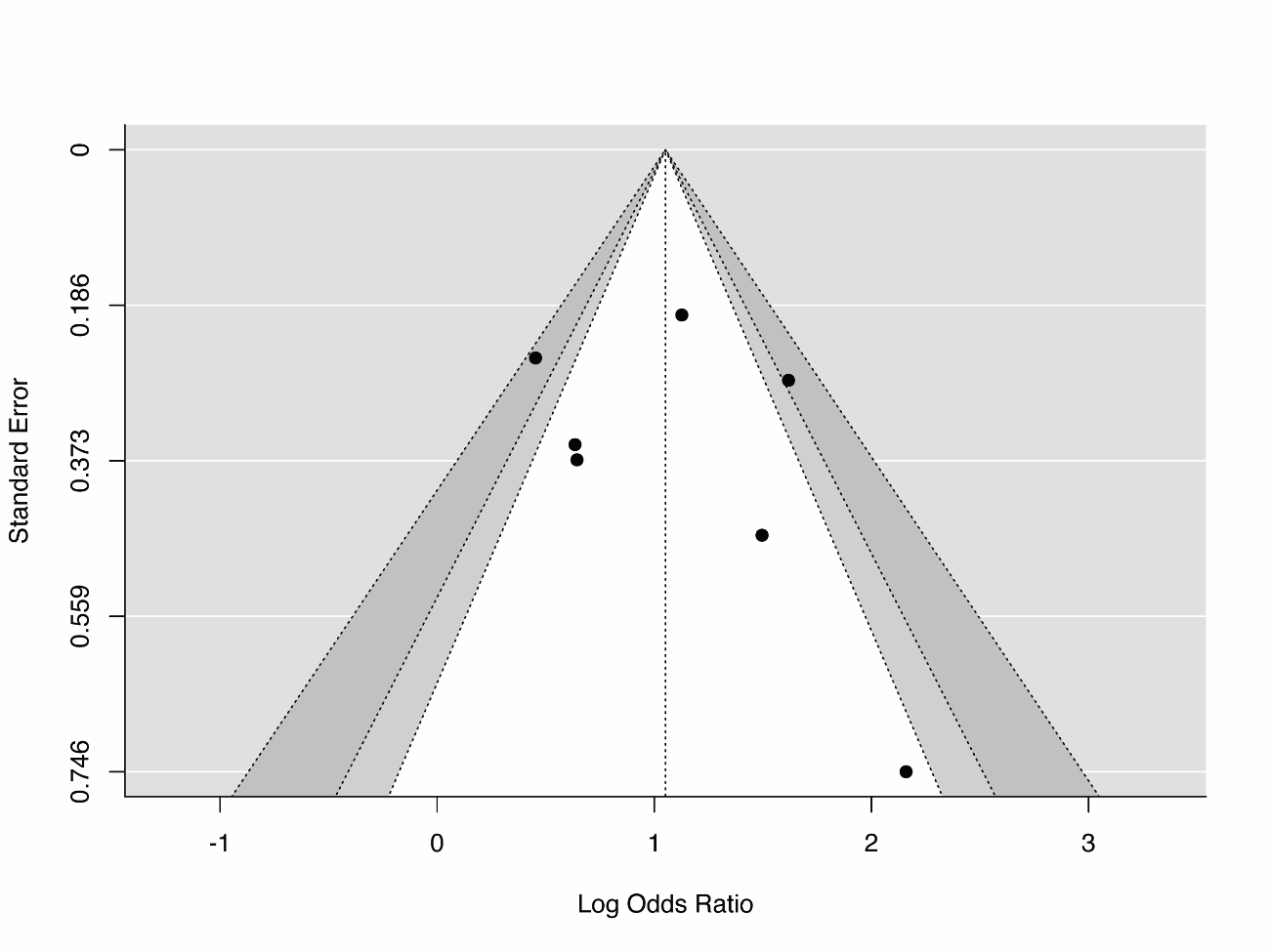


Figure B: Forest plot of association between chronic exposure to fluoride and cognitive deficit after sensitivity analysis.
